# Circulation and Respiration. Second Series of Collected Papers

**Published:** 1916-12

**Authors:** 


**Circulation and Respiration. Second Series of Collected Papers.** By Sir Thomas Lauder Brunton, Bart., M.D., D.Sc. Pp. xxi. 719. Illustrated. London : Macmillan & Co. Limited. 1916. Price 5s. net.

Many of these papers are old and valued contributions, while the more recently written are representative of the later researches and teachings of one of the most distinguished and scientific physicians, who. though no longer in the flesh, will live for many years in the spirit of medical science. Medical libraries without these papers are incomplete, and though for the most part the contents of this volume are no doubt already on their shelves in the various journals in which they were first published, the convenience for purposes of reference of the collected series, published in book form, will be appreciated far and wide.

